# Harnessing AI for Hypothyroidism Monitoring Using Wearable-Derived Heart Rate Data: Retrospective Cohort Study

**DOI:** 10.2196/86030

**Published:** 2026-10-28

**Authors:** Kyubo Shin, Yewon Jung, Won Sang Yoo, Min Joo Kim, Tae Jung Oh, Joon Ho Moon, Sung Hye Kong, Won Woo Lee, Jae Hoon Moon

**Affiliations:** 1 Thyroscope Inc, Ulsan, Republic of Korea; 2 Department of Internal Medicine, Dankook University College of Medicine, Cheonan, Republic of Korea; 3 Department of Internal Medicine, Seoul National University Bundang Hospital and Seoul National University College of Medicine, 82, Gumi-ro 173 Beon-gil, Bundang-gu, Seongnam, 13620, Republic of Korea, 82 317877068; 4 Department of Nuclear Medicine, Seoul National University Bundang Hospital and Seoul National University College of Medicine, Seongnam, Republic of Korea; 5 Center for Artificial Intelligence in Healthcare, Seoul National University Bundang Hospital, Seongnam, Republic of Korea

**Keywords:** hypothyroidism, artificial intelligence, machine learning, wearable device, heart rate

## Abstract

**Background:**

Hypothyroidism is a prevalent endocrine disorder characterized by an insidious onset and nonspecific symptoms, often leading to significant diagnostic delays. While biochemical thyroid function tests (TFTs) are the gold standard, their invasive nature and cost make them impractical for continuous, proactive screening of at-risk populations. Thyroid hormones exert direct chronotropic effects on the cardiovascular system, and their levels are closely correlated with heart rate (HR). Wearable devices capable of continuous HR monitoring offer a novel opportunity for noninvasive, digital phenotype–based detection.

**Objective:**

This study aimed to develop and validate an AI-assisted system that uses wearable-derived sleep HR data as a digital monitoring tool for individuals at risk of hypothyroidism, helping identify those who warrant further confirmatory thyroid testing.

**Methods:**

This retrospective cohort study used 1677 paired observations consisting of sleep HR data and serum TFT results, including free thyroxine (T4) and thyroid-stimulating hormone levels, from 413 participants (mean age 43.1, SD 9.7 y; women: n=314, 76%). Participants wore consumer-grade wearables (Fitbit) that recorded HR and sleep data. Each TFT result was paired with sleep HR data extracted from the 15 consecutive nights preceding the blood test. An AI system was developed using a gradient boosting algorithm (LightGBM) to classify thyroid status by integrating baseline TFT results with statistical and temporal HR features. The model’s performance was evaluated using patient-level leave-one-out cross-validation. To examine the benefit of longitudinal data, performance was compared using 1, 2, or 3 prior paired observations as reference points.

**Results:**

To ensure clinical relevance and avoid performance inflation from hyperthyroid cases, we prioritized the analysis of a subset containing only euthyroid and hypothyroid data (n=744 paired observations). In this clinically challenging subset, the system achieved an accuracy of 74.6%, with a sensitivity of 82% and a specificity of 73.5% using a single-reference data pair. Incorporating longitudinal HR patterns further enhanced performance; using 3 reference pairs improved accuracy to 78.1% (sensitivity 81.8% and specificity 77.7%). In the overall dataset (n=1677 paired observations), the system demonstrated an accuracy of 80.8%, with a sensitivity of 82% and a specificity of 80.8% using a single-reference pair. Notably, the system achieved high clinical safety by detecting 96.3% of severe overt hypothyroidism cases (free T4 <0.5 ng/dL).

**Conclusions:**

This AI-assisted system demonstrates the feasibility of a sensitive, noninvasive longitudinal monitoring tool for hypothyroidism, robustly identifying a high proportion of hypothyroid cases—particularly severe ones—to prioritize at-risk individuals for confirmatory blood tests. Given the predominantly female iatrogenic-withdrawal cohort, prospective sex-balanced validation in primary hypothyroidism is warranted.

## Introduction

Hypothyroidism is a common endocrine disorder [1,2] for which diagnosis is often significantly delayed due to its insidious onset and nonspecific symptoms, such as fatigue, weight gain, and cold intolerance. This delay means many individuals endure a diminished quality of life before receiving treatment, placing a substantial burden on both patients and the health care system [3,4]. The current diagnostic pathway relies entirely on biochemical thyroid function tests (TFTs) from blood samples, a method that is impractical for screening every person who presents with ambiguous symptoms [4,5]. Consequently, there is a significant clinical need for a sensitive and accessible monitoring and secondary screening tool to help identify at-risk individuals who would most benefit from definitive laboratory testing.

The emergence of wearable devices, which continuously monitor real-time biosignals such as heart rate (HR), offers a promising solution to this challenge [6,7]. Thyroid hormones exert direct effects on the cardiovascular system, and their levels are closely correlated with resting HR [8]. Our research group has previously established the strong association between HR from wearables and serum thyroxine levels in patients with thyrotoxicosis [9,10], successfully developing an AI-based system for its prediction [11]. However, developing a predictive system for hypothyroidism is particularly challenging because its physiological changes are more subtle and variable than those seen in thyrotoxicosis. Unlike the overt tachycardia often exceeding 100 bpm in thyrotoxic states, hypothyroidism typically results in a modest bradycardic shift. These changes often remain within the conventionally defined normal resting HR range (60‐100 bpm), making them clinically inconspicuous and highly variable among individuals. Although our previous research demonstrated a correlation between resting HR and thyroid hormone levels in patients with hypothyroidism, the degree of change was modest, leading to a lower signal-to-noise ratio [12]. This challenge illustrates the limitation of single-metric approaches and emphasizes the need for more sophisticated modeling. We hypothesized that a machine learning system, capable of learning the complex, nonlinear patterns from longitudinal HR data, could achieve high sensitivity in detecting the faint digital phenotype of hypothyroidism—defined here as the subtle, low-amplitude downward shift in HR distribution that is frequently obscured by the noise of daily activities and lifestyle factors.

This study, therefore, aimed to develop and validate an AI-assisted system using sleep HR data from wearable devices, not as a primary screening tool for the general public but as a longitudinal monitoring and secondary screening tool for at-risk populations. Our goal was to create a system capable of identifying a large proportion of individuals with undiagnosed hypothyroidism, thereby prioritizing them for confirmatory testing and enabling earlier clinical intervention.

## Methods

### Study Design and Participants

This retrospective cohort study integrated data from 5 clinical studies conducted at Seoul National University Bundang Hospital (SNUBH) to investigate the relationship between thyroid function and biosignals collected from wearable devices. The included datasets comprised (1) a proof-of-concept study evaluating the feasibility of resting HR monitoring in patients with thyrotoxicosis, (2) a proof-of-concept study assessing resting HR monitoring in patients with hypothyroidism, (3) a follow-up data collection study that recruited additional patients with thyrotoxicosis, (4) a follow-up data collection study that enrolled additional patients with hypothyroidism, and (5) a clinical study evaluating the clinical utility and usability of a mobile app for HR monitoring in patients with thyrotoxicosis. Participants in the hypothyroidism-related studies were adults aged 20 to 60 years who had undergone total thyroidectomy for differentiated thyroid cancer and were scheduled for radioactive iodine (RAI) scanning. As part of these clinical protocols, thyroid hormone therapy had been temporarily withdrawn to induce iatrogenic hypothyroidism, and TFTs had been performed at 3 time points: during hormone therapy, 1 month after withdrawal, and 1 month after resumption. Participants in the thyrotoxicosis-related studies were adults aged ≥20 years who had been newly diagnosed with or experienced a recurrence of thyrotoxicosis and were undergoing antithyroid drug therapy. The inclusion criteria for these studies were consistent with those used in the original proof-of-concept study by Lee et al [9,10], with a minor modification to the age requirement. Although the eligibility criterion for the thyrotoxicosis-related studies permitted enrollment of adults aged >60 years, no such participant was enrolled; the pooled cohort therefore comprised adults aged 20 to 60 years. Across all integrated studies, participants using medications that directly influence HR (eg, long-acting β-blockers and calcium channel blockers) were strictly excluded. The only exception was the permitted use of short-acting β-blockers in thyrotoxicosis cohorts for temporary symptom relief. This approach mirrors real-world management, as our prior validation showed that intermittent daytime use of such medications does not significantly alter sleep HR-based AI performance [11]. All participants were required to own a smartphone and be capable of using a wearable device and its associated mobile app. During the study period, wearable devices continuously collected HR, sleep, and activity data.

### Ethical Considerations

This study was conducted in accordance with the ethical standards of the Institutional Review Board (IRB) of SNUBH and the Declaration of Helsinki. This study used data from 5 primary clinical studies, each independently approved by the SNUBH IRB under the following application numbers: B-1609-363-004, B-1906-547-304, B-2012-654-303, B-2209-783-301, and B-2201-735-304. The secondary analysis of the integrated datasets was reviewed and approved by the SNUBH IRB as an amendment to the protocol of the primary study approved under case B-2209-783-301; this application number therefore appears both among the primary study approvals and as the approval covering the present secondary analysis.

Informed consent had been obtained from all participants prior to their enrollment in the original studies, which included consent for the use of biosignal and clinical data for subsequent research purposes. For the current retrospective secondary analysis, all data were strictly deidentified and anonymized before analysis to ensure participant privacy and confidentiality. Participant data were handled in accordance with institutional guidelines for protecting personal health information. No additional compensation was provided to participants for this retrospective secondary analysis beyond what was provided in the primary studies. Furthermore, we confirm that all representative patient data shown in the figures have been fully deidentified, and no individual participants can be identified.

### Wearable Devices and Apps

Across the 5 studies included in this analysis, various models of Fitbit wearable devices and the Fitbit mobile app were used to collect HR, physical activity, and sleep data. The following devices and configurations were used:

- In the proof-of-concept study for hypothyroidism, the Fitbit Charge 2 (Fitbit Inc) was used in conjunction with the Fitbit app for iOS (Apple Inc) or Android (Google LLC). The firmware version at the end of the study was 22.58.0.
- In the follow-up data collection study for hypothyroidism, the Fitbit Inspire 3 was used with the Fitbit app for iOS or Android. The firmware version at the end of the study was 63.20001.188.58.
- In the proof-of-concept study for thyrotoxicosis, participants used either the Fitbit Charge HR or Fitbit Charge 2. The firmware versions were 18.128 for the Charge HR and 22.53.4 for the Charge 2 at the end of the study.
- In the follow-up data collection study for thyrotoxicosis and the usability and utility study, participants used either the Fitbit Inspire or Fitbit Inspire 2. The firmware versions were 1.88.11 for the Inspire and 1.124.28 for the Inspire 2 at the end of the study.

All Fitbit devices used across these studies shared the same core sensors and algorithms for measuring physiological signals. Physical activity was recorded using a 3-axis accelerometer, while HR was measured via photoplethysmography (PPG). Fitbit’s proprietary algorithms aggregate the raw PPG signals internally and expose HR values at a maximum temporal resolution of 1 sample per minute through the Fitbit Web API; accordingly, all sleep HR analyses in this study were conducted at a 1-minute resolution. Fitbit’s proprietary algorithms also provided sleep-related metrics, including total sleep duration, number of awakenings, and time awake, by integrating accelerometer and HR data. To collect both interday summary data (activity, HR, and sleep) and intraday detailed data (activity and HR), we used Glandy (Thyroscope Inc), a mobile self-management app for thyroid dysfunction. The app, available for both iOS and Android platforms, was connected to Fitbit via its official API, allowing secure and authorized data transfer following user consent.

### Biochemical Measurements

Serum concentrations of free T4 and thyroid-stimulating hormone (TSH) were measured using immunoradiometric assays (free T4: DiaSorin S.p.A.; TSH: CIS Bio International). The analytical sensitivities were 0.05 ng/dL for free T4 and 0.04 mIU/L for TSH, with a functional sensitivity of 0.07 mIU/L for the TSH assay. Reference ranges were defined as 0.89 to 1.78 ng/dL for free T4 and 0.3 to 4.0 mIU/L for TSH. Thyroid function status was determined based on these laboratory results. Euthyroid status was defined as both free T4 and TSH levels being within the reference range. Overt hypothyroidism was defined as a free T4 level below the reference range accompanied by an elevated TSH level, whereas subclinical hypothyroidism was defined as a normal free T4 level with elevated TSH. In contrast, overt hyperthyroidism was defined as a free T4 level above the reference range with suppressed TSH, and subclinical hyperthyroidism was defined as a normal free T4 level with suppressed TSH.

### Data Preparation

The dataset was constructed using “HR-TFT pairs,” in which each TFT result—typically obtained during a daytime clinical visit—was linked to 1-minute resolution sleep HR data recorded on the 15 consecutive nights preceding the test. Sleep HR was selected as the primary biosignal because it represents a period of relative autonomic stability, minimizing confounding from psychological stress, physical activity, caffeine intake, and other daytime lifestyle factors. This allowed the AI system to capture thyroid-related physiological signals with a higher signal-to-noise ratio than active-state HR. Sleep HR data were aligned to the date on which each sleep session ended, and HR values were extracted from periods identified as sleep by the Fitbit device. These data consisted of detailed intrasleep HR measurements, matched to each individual’s sleep window. To account for real-world compliance variability and ensure the system’s robustness, a 15-day window was included in the analysis if it contained at least one night with a recorded sleep duration of ≥3 hours. Missing data or skipped nights within the window were not imputed. Instead, all statistical and temporal features were computed independently for each valid sleep session using the 1-minute resolution HR measurements within that session (≥3 h×60 min=≥180 measurements per session, substantially exceeding the minimum sample requirement of any feature in our set, including the seasonal lag-1 autocorrelation function feature, which requires ≥72 observations). The median value across all valid sessions within the 15-day window was then used as the final feature value for model input, ensuring mathematical robustness while accommodating the variability in valid-night counts inherent to wearable usage in real-world settings. This filtering process ensured that the AI model learned from authentic physiological patterns while accommodating the practicalities of long-term wearable use. We further distinguished between training and test data conditions. Training used the HR-TFT pairs presented in Table 1 as the underlying data source, including both forward and reverse temporal orderings of reference-target combinations during model fitting. In contrast, test data (used for the performance evaluations reported in Tables 2 and 3) were restricted to observations with same-day sleep HR data on the target date and used only forward temporal orderings, reflecting the intended deployment scenario in which the system predicts hypothyroid status on the day following the availability of sleep HR data given prior reference HR-TFT pairs. As a single target HR-TFT pair can be paired with multiple different prior reference pairs from the same participant, the number of prediction outputs generated for evaluation is not equal to the number of HR-TFT pairs; the reconciliation between the raw HR-TFT pair counts of Table 1 and the reference-configuration output counts of Tables 2 and 3 is provided in Multimedia Appendix 1 (state-wise test-cohort breakdown) and Multimedia Appendix 2 (reference-target pair counts and output counts per configuration).

**Table 1.** Participant and data characteristics (413 participants and 1677 paired HR^a^-TFT^b^ observations).

Characteristic	Value
Age (years), mean (SD)	43.1 (9.7)
Sex, n (%)	
Male	99 (24)
Female	314 (76)
Thyroid function state of data	
Overt hyperthyroidism (n=344 pairs^c^)	
Free T4^d^ (ng/dL), mean (SD)	2.67 (1.48)
TSH^e^ (mIU/L)	<0.05
Subclinical hyperthyroidism (n=589 pairs^c^)	
Free T4 (ng/dL), mean (SD)	1.38 (0.24)
TSH (mIU/L), median (IQR)	0.03 (0.03-0.05)
Euthyroid (n=485 pairs^c^)	
Free T4 (ng/dL), mean (SD)	1.23 (0.19)
TSH (mIU/L), median (IQR)	1.36 (0.85-1.95)
Subclinical hypothyroidism (n=110 pairs^c^)	
Free T4 (ng/dL), mean (SD)	1.17 (0.22)
TSH (mIU/L), median (IQR)	6.65 (5.30-16.26)
Overt hypothyroidism (n=149 pairs^c^)	
Free T4 (ng/dL), mean (SD)	0.49 (0.29)
TSH (mIU/L), median (IQR)	33.98 (2.17-63.97)

a HR: heart rate.

b TFT: thyroid function test.

c Number of paired HR-TFT observations classified as that state at the time of the blood test.

d Free T4: free thyroxine.

e TSH: thyroid-stimulating hormone.

**Table 2.** Performance of the AI-assisted system for detecting hypothyroidism in euthyroid and hypothyroid subset.

Number of reference data^a^	Accuracy (%)	Sensitivity (%)	Specificity (%)	PPV^b^ (%)	NPV^c^ (%)	Sensitivity (%) for free T4^d^ levels (ng/dL)		
						Free T4<0.5	0.5≤free T4<0.7	0.7≤free T4<0.89
1 (n=803)	74.6	82.0	73.5	30.6	96.6	96.3	81.3	75.4
2 (n=204)	77.0	84.0	76.0	32.8	97.1	100	100	76.5
3 (n=105)	78.1	81.8	77.7	30.0	97.3	100	100	71.4

a Values in parentheses (n) indicate the number of prediction outputs generated under each reference configuration on the euthyroid-hypothyroid test cohort. Each output corresponds to a single target-date classification produced by the AI-assisted system using the specified number of prior reference heart rate (HR)-thyroid function test (TFT), pairs from the same participant; the number of underlying reference-target pairs used as model input equals the number of references×the number of outputs. This cohort excludes hyperthyroid states from Table 1 and comprises forward-ordered reference-target combinations whose target date has same-day sleep HR data, as required by the deployment scenario. As a single target date may pair with multiple prior reference dates, a target date can contribute more than 1 output, and output counts are therefore not a strict subset of the Table 1 pair counts (the 803 single-reference outputs arise from 352 unique target dates; a sensitivity analysis restricted to 1 output per unique target date is reported in the “Results” section). See Multimedia Appendix 1 for the state-wise test-cohort breakdown and Multimedia Appendix 2 for the reference-configuration counts.

b PPV: positive predictive value.

c NPV: negative predictive value.

d Free T4: free thyroxine.

**Table 3.** Overall performance of AI-assisted system for detecting hypothyroidism^a^.

Number of reference data^a^	Accuracy (%)	Sensitivity (%)	Specificity (%)	PPV^b^ (%)	NPV^c^ (%)
1 (n=1695)	80.8	82.0	80.8	21.1	98.6
2 (n=438)	82.9	84.0	82.8	22.83	98.8
3 (n=221)	84.6	81.8	84.8	21.95	98.9

a Values in parentheses (n) indicate the number of prediction outputs generated under each reference configuration on the overall test cohort. Each output corresponds to a single target-date classification produced by the AI-assisted system using the specified number of prior reference heart rate (HR)-thyroid function test (TFT) pairs from the same participant; the number of underlying reference-target pairs used as model input equals the number of references×the number of outputs. The test cohort comprises forward-ordered reference-target combinations of the HR-TFT pairs in Table 1, restricted to combinations whose target date has same-day sleep HR data, as required by the deployment scenario. Because a single target date may pair with multiple prior reference dates, a target date can contribute more than 1 output, and output counts are therefore not a strict subset of the Table 1 pair counts (the 1695 single-reference outputs arise from 753 unique target dates; a sensitivity analysis restricted to 1 output per unique target date is reported in the “Results” section). See Multimedia Appendix 1 for the state-wise test-cohort breakdown and Multimedia Appendix 2 for the reference-configuration counts.

b PPV: positive predictive value.

c NPV: negative predictive value.

### Development of the AI-Assisted System

The proposed system is designed to classify whether a user has hypothyroidism using the relationship between the change in serum free T4 level and the change in HR data, where the relationship is represented by the data combining 2 HR-TFT pairs (one for the date of the referred TFT and another for the target date) of a user. The input of the system consists of the results of the referred TFT (ie, serum free T4 level) and the change in HR data between the 2 different time points. The binary output indicates whether the individual is hypothyroid at the target date. Data with a free T4 level <0.89 ng/dL at the target date were labeled as positive (hypothyroid state).

The input features used in the system can be categorized into 3 types: the laboratory results from the referred TFT, statistical characteristics of HR distributions, and temporal features capturing the sequential structure of HR data. These HR-based features are extracted independently from both the referred TFT date and the target date. The model incorporates both the absolute values of these features at each time point and the differences between them. While the temporal differences help capture the dynamic relationship between HR patterns and changes in free T4 concentration, the individual time point features allow the model to leverage their direct correlation with free T4 levels. To effectively capture the nonlinear relationship between HR dynamics and thyroid function, we used gradient boosting algorithms (LightGBM) in constructing the classification system. To address the heterogeneity in sleep HR distributions across individuals, which can reduce model convergence when treated as a single population, the classifier was implemented as 2 parallel LightGBM submodels: a P submodel trained on observations with a target mean sleep HR of ≥62.5 bpm, and an M submodel trained on observations with a target mean sleep HR <62.5 bpm. Each observation was routed to the corresponding submodel based on its target mean sleep HR, and the 2 submodels produced independent predictions for their respective subsets. A schematic overview of the AI-assisted system is presented in Figure 1. The final curated input set comprises 26 features—19 used in the P submodel and 14 in the M submodel, of which 7 are shared—and the candidate feature families, their mathematical definitions, and the full enumeration of the 26 selected features are provided in Multimedia Appendix 3. LightGBM was selected after benchmarking against logistic regression and random forest under identical participant-level 10-fold cross-validation conditions using the full pool of 107 candidate features without prior feature selection and applying balanced class weighting to all models. Under these fair-comparison conditions, LightGBM achieved the highest AUROC (0.695 vs 0.687 for random forest and 0.664 for logistic regression). Although logistic regression achieved a marginally higher area under the PR curve (AUPRC; 0.139 vs 0.118 for LightGBM)—likely reflecting the precision-recall (PR) behavior of L1-penalized linear models on the full 107-feature pool—LightGBM was selected based on its threshold-independent discriminative superiority (AUROC), its capacity to model nonlinear physiological interactions inherent in the HR–thyroid hormone relationship, native handling of missing values in wearable data, and compatibility with Shapley Additive Explanations (SHAP)–based interpretability of feature contributions (Multimedia Appendix 4). The 10-fold benchmarking framework was designed for relative algorithm comparison under fair conditions and is not directly comparable to the leave-one-out cross-validation (LOOCV)–based primary analysis using the curated 26-feature set (Tables 2 and 3).

**Figure 1.**
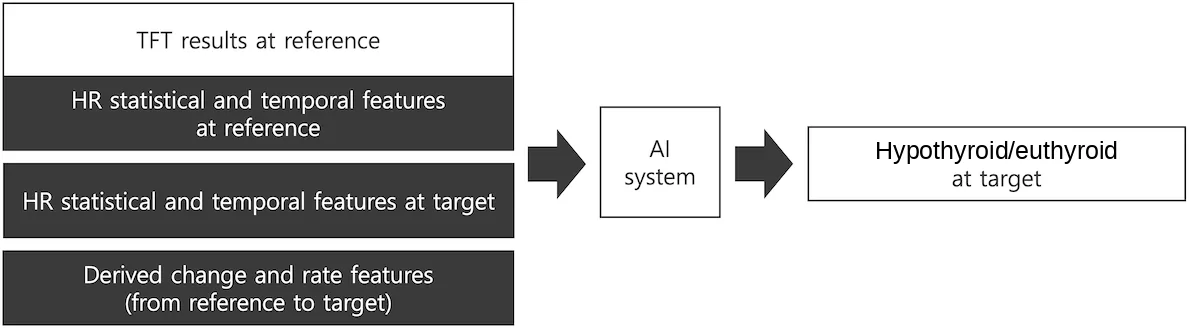
Schematic overview of the AI-assisted system for monitoring thyroid function status using wearable-derived heart rate data. The system integrates thyroid function test (TFT) results and heart rate (HR) statistical and temporal features obtained at the reference time point with HR features measured at the target time point. Derived change and rate features between reference and target are also included as inputs. Using these features, the AI-assisted system predicts the thyroid function state (hypothyroid or euthyroid) at the target time point.

### Statistical Analysis

The diagnostic performance of the AI-assisted system for detecting hypothyroidism was evaluated by calculating sensitivity, specificity, positive predictive value (PPV), and negative predictive value (NPV). Receiver operating characteristic (ROC) curves were constructed, and the area under the curve (AUC) was computed to quantify the model’s overall discriminative ability. To complement the ROC analysis under the class imbalance inherent in our cohort, PR curves were additionally constructed, and the AUPRC was computed. As PR curves are sensitive to the prevalence of the positive class, they provide a more informative view of classifier performance in imbalanced settings than ROC curves alone. For each configuration, the prevalence of hypothyroidism served as the baseline reference for a noninformative classifier. The AI model was evaluated while varying the number of reference datasets used as input (1, 2, or 3) to examine how multiple reference inputs affected predictive performance. Model evaluation was conducted using a modified LOOCV strategy, in which all data from a single participant were held out as the test set while the model was trained on data from the remaining participants. No model architecture or hyperparameters were adjusted within the cross-validation procedure; the same parameters were applied consistently across all folds. Feature selection was performed once on the entire dataset using a greedy forward selection strategy guided by cross-validation AUC prior to the final participant-level LOOCV evaluation. This feature selection step was therefore not fully nested within each LOOCV fold. All hyperparameters (n_estimators=200, num_leaves=15, learning_rate=0.1) were fixed prior to the formal analysis based on established defaults in the literature and confirmed through preliminary experiments and were not adjusted during cross-validation. This patient-level approach was designed to assess the system’s generalizability to previously unseen individuals. Furthermore, this patient-level cross-validation design ensures that any overlapping HR windows from the same participant—which were rare in practice (median interval between consecutive blood tests within the same participant 33, IQR 28‐42 days; only 1.6% of consecutive test intervals were shorter than the 15-day window length)—are always held out together as the test fold, eliminating the principal statistical concern regarding nonindependence of overlapping observations between training and test sets. In addition, because a single target date can pair with multiple prior reference dates and thereby contribute more than one prediction output within a reference configuration, a sensitivity analysis was performed in which exactly 1 output per unique target date was retained—selecting the output based on the most recent prior reference, mirroring the intended deployment scenario—and all performance metrics were recomputed. To interpret the contribution of each input feature to the model’s predictions, we performed a post hoc feature importance analysis using SHAP. SHAP values were computed separately for the P and M submodels using the TreeExplainer, and the mean absolute SHAP value of each feature was used to summarize its overall contribution to model output. Continuous variables with a normal distribution are expressed as mean (SD), while those with a nonnormal distribution are presented as median (IQR). All statistical analyses and model evaluations were performed using Python (version 3.11; Python Software Foundation).

## Results

### Participant and Data Characteristics

A total of 1677 HR-TFT pairs were collected from 413 participants (mean age 43.1, SD 9.7 years; men: n=99, 24%; and women: n=314, 76%). On the basis of corresponding TFTs, the dataset included 344 overt hyperthyroidism, 589 subclinical hyperthyroidism, 485 euthyroid, 110 subclinical hypothyroidism, and 149 overt hypothyroidism cases. The mean serum free T4 and TSH levels for each group are summarized in Table 1. In particular, the overt hypothyroidism group exhibited markedly low free T4 (mean 0.49, SD 0.29 ng/dL) and elevated TSH levels (median 33.98, IQR 2.17-63.97 mIU/L).

### Performance in the Euthyroid and Hypothyroid Subset

To evaluate the system’s ability to detect the transition from a euthyroid to a hypothyroid state—the most clinically relevant scenario—we first analyzed the subset excluding hyperthyroid cases (744 HR-TFT pairs, yielding 803 single-reference prediction outputs; Table 2). In this subset, the system achieved an accuracy of 74.6%, with a sensitivity of 82% and a specificity of 73.5% when using a single-reference HR-TFT pair. The system also demonstrated strong performance in detecting severe overt hypothyroidism (free T4 <0.5 ng/dL), achieving a sensitivity of 96.3%. Stratified sensitivity for milder hypothyroid states showed good performance as well, with 75.4% sensitivity for free T4 between 0.7 and 0.89 ng/dL and 81.3% for free T4 between 0.5 and 0.7 ng/dL. When multiple reference pairs were applied, accuracy further improved to 78.1%, with a sensitivity of 81.8% and specificity of 77.7%. This trend was also evident in stratified analyses. Specifically, when using 3 reference pairs, the model achieved 100% sensitivity in detecting cases with free T4 <0.7 ng/dL.

### Overall Performance of the AI-Assisted System for Detecting Hypothyroidism

We further evaluated the model on the overall dataset (1677 HR-TFT pairs, yielding 1695 single-reference prediction outputs) to confirm its ability to distinguish hypothyroidism across the full thyroid function spectrum (Table 3). In the overall cohort, accuracy was 80.8%, with a sensitivity of 82% and specificity of 80.8%. This higher accuracy was partially attributable to the AI’s consistent ability to correctly classify all overt and subclinical hyperthyroid cases as negative (nonhypothyroid). When multiple reference HR-TFT pairs were used to assess a target HR dataset, the model’s predictive performance improved. With 2 reference pairs, overall accuracy increased to 82.9%, and with 3 reference pairs, it further improved to 84.6%. At this level, the model achieved a sensitivity of 81.8%, a specificity of 84.8%, and an NPV of 98.9%.

### ROC and PR Analyses

ROC curve analysis was performed to further evaluate the discriminative performance of the AI-assisted system across different reference configurations and thyroid function states (Figure 2). In the subset including only euthyroid and hypothyroid cases, the system demonstrated robust performance, yielding AUC values of 0.7931, 0.8317, and 0.7998 when 1, 2, and 3 reference datasets were used, respectively (Figure 2A). Similarly, in the overall cohort, the AUC was 0.8455, 0.8697, and 0.8489 for the corresponding configurations, with the highest AUC observed when 2 references were applied (Figure 2B).

**Figure 2.**
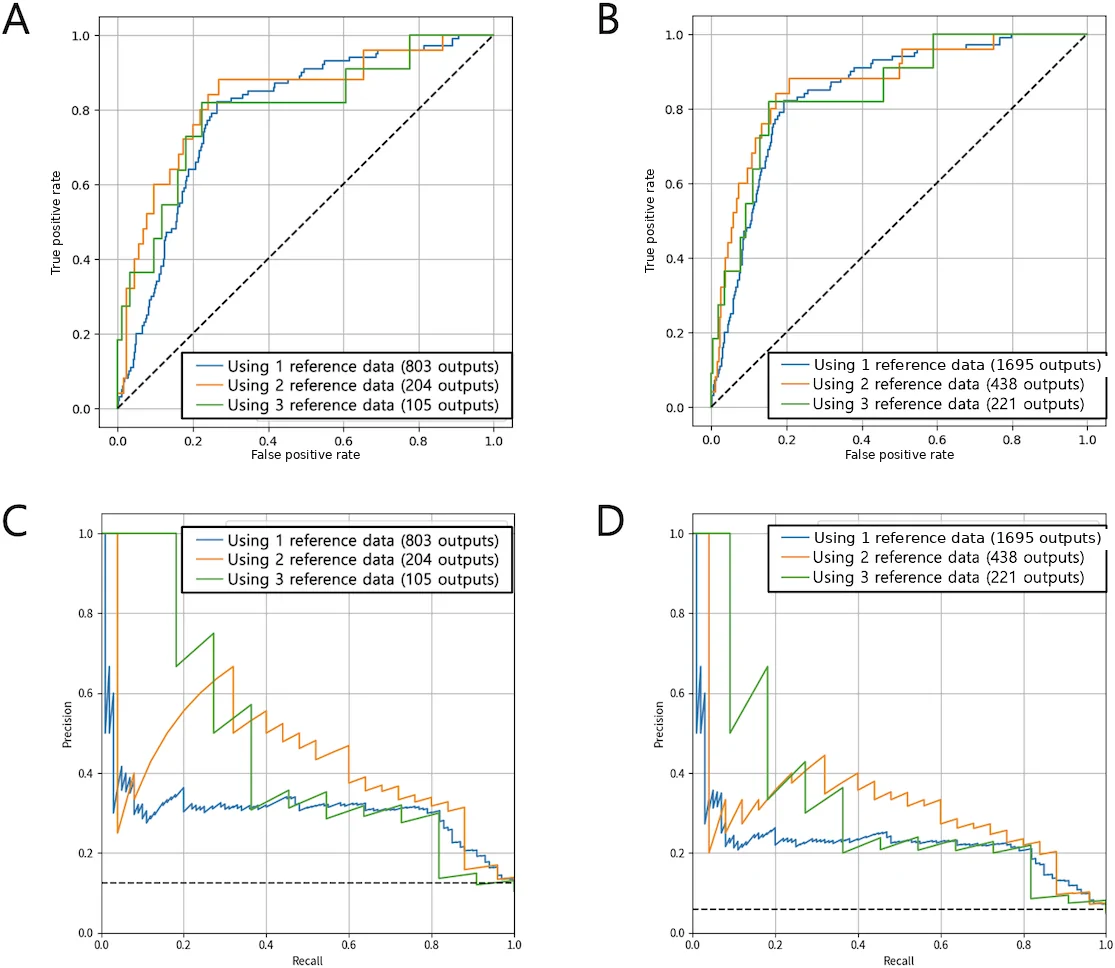
Receiver operating characteristic (ROC) and precision-recall (PR) curves of the AI-assisted system for detecting hypothyroidism. (A) ROC curves in the euthyroid-hypothyroid subset (n=803 prediction outputs at the single-reference configuration) using 1, 2, or 3 reference heart rate–hyroid function test pairs; area under the ROC curve (AUC)=0.7931, 0.8317, and 0.7998, respectively. (B) ROC curves in the overall test cohort (n=1695 prediction outputs at the single-reference configuration) under the corresponding reference configurations; AUC=0.8455, 0.8697, and 0.8489. (C) Precision-recall (PR) curves in the euthyroid-hypothyroid subset; area under the PR curve (AUPRC)=0.3142, 0.4490, and 0.4773 compared with a baseline prevalence of 0.125 (100/803). (D) PR curves in the overall test cohort; AUPRC=0.2329, 0.3269, and 0.3449 compared with a baseline prevalence of 0.059 (100/1695).

To further evaluate performance under class imbalance, PR curves were constructed for the same reference configurations (Figures 2C and 2D). In the euthyroid-hypothyroid subset, AUPRC values were 0.3142, 0.4490, and 0.4773 for 1, 2, and 3 reference datasets, respectively, substantially exceeding the baseline prevalence of approximately 0.125 (100/803 at the single-reference configuration; Figure 2C). In the overall cohort, AUPRC values were 0.2329, 0.3269, and 0.3449 for the corresponding configurations, compared to the baseline prevalence of approximately 0.059 (100/1695 at the single-reference configuration; Figure 2D). The fact that AUPRC values remained markedly above the prevalence baseline in both cohorts indicates that the AI-assisted system preserves a clinically meaningful PR trade-off despite the imbalance in the overall dataset.

### Sensitivity Analysis Restricted to Unique Target Dates

As a single target date can pair with multiple prior reference dates, individual target dates may contribute more than 1 prediction output within a reference configuration. In the single reference configuration, the 1695 outputs in the overall test cohort arose from 753 unique target dates (1‐14 reference pairings per target date; mean 2.25; 315 target dates had exactly 1 available reference), and the 803 outputs in the euthyroid-hypothyroid cohort arose from 352 unique target dates. The 100 positive outputs, identical in both cohorts, arose from 47 unique positive target dates. Of the 1677 HR-TFT pairs in Table 1, 753 served as target dates; the remaining 924 pairs lacked same-day sleep HR data on the target date or a prior reference from the same participant and therefore did not serve as targets, although many contributed as reference dates. The repetition amounts to 942 (55.6%) of the 1695 outputs being additional appearances of a target date beyond its first. Restricting the evaluation to 1 output per unique target date—retaining the output based on the most recent prior reference—yielded performance metrics materially unchanged from the primary analysis. In the overall cohort (38 true positives, 9 false negatives, 563 true negatives, and 143 false positives), accuracy was 79.8% (601/753; vs 80.8% in the primary analysis), sensitivity was 80.9% (38/47; vs 82.0%), specificity was 79.7% (563/706; vs 80.8%), PPV was 21% (38/181; vs 21.1%), and NPV was 98.4% (563/572; vs 98.6%). In the euthyroid-hypothyroid cohort (38 true positives, 9 false negatives, 221 true negatives, and 84 false positives), accuracy was 73.6% (259/352; vs 74.6%), sensitivity was 80.9% (38/47; vs 82.0%), specificity was 72.5% (221/305; vs 73.5%), PPV was 31.1% (38/122; vs 30.6%), and NPV was 96.1% (221/230; vs 96.6%). All differences were within 1.1 percentage points, indicating that the contribution of multiple outputs per target date did not materially inflate the reported performance.

### Sex-Stratified Performance

To assess whether the system performs equitably across sexes, we conducted a sex-stratified performance analysis. The model demonstrated comparable specificity (men: 165/198, 83.3%; women: 1123/1397, 80.4%) and nearly identical PPV (men: 9/42, 21.4%; women: 73/347, 21.0%) across sexes. Although sensitivity was lower in men (9/14, 64.3%) than in women (73/86, 84.9%), the male subgroup contained only 14 positive observations from 7 participants, compared to 86 positive observations from 33 female participants. With fewer than 30 positive male observations, the male sensitivity estimate is statistically unstable—the classification of even a single case shifts the estimate by approximately 7 percentage points—and does not permit a reliable assessment of performance disparity between sexes.

### Feature Importance Analysis

To clarify which input features most influenced the classifier’s output, we examined the mean absolute SHAP values of each feature for both submodels (Figure 3). In the P submodel (target mean sleep HR ≥62.5 bpm), spikiness of the target-date sleep HR series was the highest-ranked feature, narrowly surpassing the laboratory feature ref_fT4 (reference serum free T4 level), followed by seasonal lag-1 autocorrelation and variance heterogeneity of the target-date HR series. In the M submodel (target mean sleep HR <62.5 bpm), ref_fT4 was the highest-ranked feature, followed by skewness, variance heterogeneity, and binarized mean of the target-date HR series. Across both submodels, features derived from the target-date HR time series consistently ranked among the top contributors alongside ref_fT4, indicating that both the reference laboratory value and wearable-derived HR dynamics meaningfully shape the classifier’s predictions.

**Figure 3.**
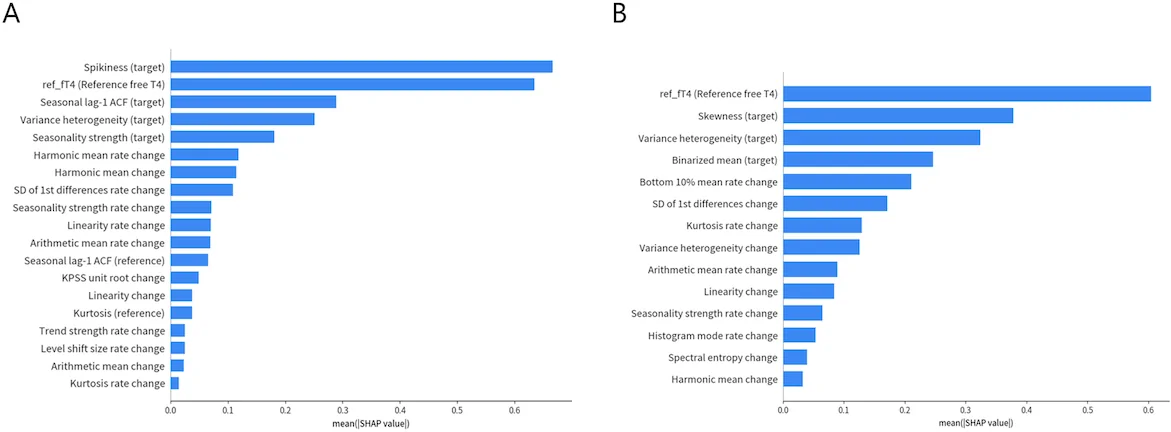
Shapley Additive Explanations (SHAP) feature importance plots for the AI-assisted hypothyroidism monitoring system. The mean absolute SHAP value of each input feature is shown, indicating the average impact of the feature on the model output. (A) P submodel (target mean sleep heart rate [HR] ≥62.5 bpm). (B) M submodel (target mean sleep HR <62.5 bpm). ACF: autocorrelation function; KPSS: Kwiatkowski-Phillips-Schmidt-Shin (test).

### Ablation Analysis of Feature Contributions

To quantify the independent contribution of wearable-derived HR features beyond the baseline laboratory value, we conducted an ablation analysis comparing 3 input configurations under identical participant-level LOOCV conditions on the overall cohort (1677 HR-TFT pairs, yielding 1695 single-reference prediction outputs) using a single-reference pair, matching the primary configuration reported in Table 3 (row 1). The full model combining ref_fT4 and HR features achieved a sensitivity of 0.820 and a specificity of 0.808; ref_fT4 alone achieved a sensitivity of 0.300 and a specificity of 0.801; and HR features alone (without ref_fT4) achieved a sensitivity of 0.470 and a specificity of 0.806. Specificity remained essentially constant across the 3 configurations (range 0.801‐0.808), so the sensitivity differential directly reflects the marginal predictive value of each feature group. Adding HR features to ref_fT4 produced a 173% relative increase in sensitivity (from 0.300 to 0.820, absolute increase of 0.52), confirming that wearable-derived HR features provide predictive information that is complementary to, and independent of, the baseline biochemical value.

## Discussion

### Principal Findings

This study evaluated the feasibility of using an AI-assisted system and wearable-derived HR data to identify hypothyroidism, a condition characterized by subtle and nonspecific physiological changes. Our findings suggest that this noninvasive approach could complement traditional biochemical testing by providing a data-driven tool for the early identification of individuals at risk. Given the higher clinical relevance, we prioritized the performance evaluation of a subset consisting solely of euthyroid and hypothyroid cases. In this clinically challenging subset, the system achieved a sensitivity of 82.0% and a specificity of 73.5% using a single-reference data pair. These metrics provide a more conservative and realistic assessment of the model’s ability to detect the transition from euthyroidism to hypothyroidism, as excluding hyperthyroid states—which present more distinct physiological signatures—makes the classification task more complex. Furthermore, the predictive accuracy for hypothyroidism increased when using longitudinal data. The overall predictive performance generally improved when integrating 2 or more reference HR-TFT data pairs compared to a single pair. The slight decrease in AUC observed with 3 reference pairs was likely due to the more limited sample size available for that specific configuration. Specifically, as the requirement for longitudinal data increased, the number of positive (hypothyroid) prediction outputs in the subset available for 3-reference evaluation decreased significantly to only 11 (33 underlying reference-target pairs). This reduction in the positive class increases statistical variance, making the AUC score more susceptible to fluctuations caused by individual prediction outcomes rather than reflecting a true decrease in model performance. Post hoc SHAP analysis further showed that wearable-derived HR features consistently ranked among the top contributors to the classifier’s predictions, alongside the reference serum free T4 level, supporting the notion that sleep HR dynamics carry information relevant to thyroid status.

A noteworthy finding regarding clinical safety is that the system demonstrated superior performance in detecting profound hormonal deficiencies. Even with a single reference pair, the AI system correctly identified 96.3% of severe overt hypothyroidism cases (free T4 <0.5 ng/dL). With the application of 2 reference pairs, the system achieved 100% accuracy for cases with free T4 levels below 0.7 ng/dL. These results indicate that the system’s occasional failures were confined to the “gray zone” (free T4 ≥0.7 ng/dL), confirming its reliability as a tool designed to prioritize patients with significant clinical risk for confirmatory biochemical testing.

### Comparison to Prior Work

Unlike the clear and dramatic cardiovascular manifestations seen in thyrotoxic states, the cardiovascular effects of hypothyroidism are often subtle and nonspecific—such as mild bradycardia or diastolic dysfunction—which by themselves are insufficiently discriminatory [13]. Moreover, recent meta-analyses confirm that while HR variability is decreased in patients with hypothyroidism, the effect sizes across time domain and frequency domain indices are modest and highly heterogeneous among individuals [14]. Although a decrease in sleep HR serves as a key differentiating factor in the hypothyroid state as seen in the raw sleep HR data and histograms from 2 representative patients in euthyroid and hypothyroid states (Figure 4), there was a modest correlation between resting HR and thyroid hormone levels in patients with hypothyroidism in our prior work [12]. The AI-assisted system developed in this study, however, achieved a significantly higher predictive performance by moving beyond linear correlations. By integrating multidimensional data streams—encompassing resting HR, variability indices, and both aggregate and time-resolved features extracted from sleep HR recordings—the model was able to learn the complex, nonlinear interactions and temporal dependencies that constitute the unique digital phenotype of hypothyroidism.

**Figure 4.**
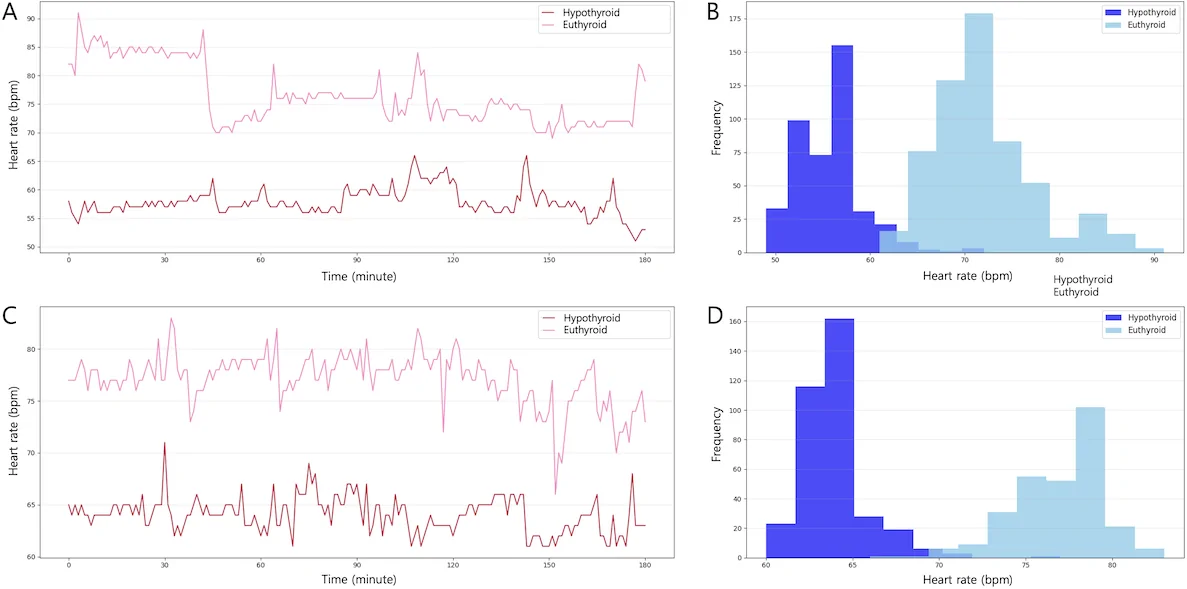
Comparison of sleep heart rate patterns between hypothyroid and euthyroid states in 2 representative patients. Sleep heart rate raw data for hypothyroid and euthyroid states (A, C) and the corresponding heart rate distribution histograms comparing the 2 states (B, D); panels A and B, and panels C and D, each represent data from the same patient.

### Clinical and Scientific Implications

Regarding clinical positioning, our system functions as a longitudinal monitoring and secondary screening tool for individuals with various thyroid vulnerabilities. Beyond subclinical hypothyroidism, this includes monitoring for iatrogenic hypothyroidism in patients receiving antithyroid medications or detecting the onset of hypothyroidism following RAI therapy. Training the AI model on a comprehensive dataset that includes hyperthyroid states was intentional, as it allows the system to learn the physiological boundaries necessary to detect these critical hormonal transitions. By incorporating a baseline (referred) TFT, the system calibrates to an individual’s unique HR–thyroid hormone relationship, shifting the paradigm from reactive, patient-initiated consultation to proactive detection. This individualized monitoring assists clinicians in prioritizing confirmatory testing, enabling earlier intervention during significant physiological shifts [14,15]. However, as the current model was trained on acute iatrogenic hormone withdrawal (median TSH 33.98 mIU/L), generalization to the slow and insidious onset of naturally occurring primary hypothyroidism requires prospective validation in dedicated cohorts. In addition, for patients already diagnosed and receiving levothyroxine replacement therapy, this technology could function as a personalized monitoring tool. By tracking physiological responses to treatment, it may offer supplementary data to clinicians for fine-tuning medication dosage, aiming for optimal patient-reported outcomes beyond simple biochemical normalization. Beyond hypothyroidism, this framework could be extended to other chronic disorders characterized by subtle physiological dysregulation, such as adrenal insufficiency or metabolic syndrome.

The SHAP analysis offers mechanistic plausibility for the model’s behavior. In both submodels, features summarizing the distributional shape and temporal structure of the target-date sleep HR series—including spikiness, skewness, variance heterogeneity, and seasonal lag-1 autocorrelation—contributed substantially to the output, alongside the reference free T4 value. This pattern is consistent with the known effects of thyroid hormone on autonomic tone and resting cardiac output, which are expected to manifest as changes in the variability and temporal structure of HR during sleep rather than as a simple shift in mean HR. Notably, HR-derived features carried predictive weight comparable to the laboratory feature ref_fT4, and in the P submodel even exceeded it. This suggests that continuously collected wearable signals could complement sparse serum measurements in tracking changes in thyroid status over time.

A post hoc breakdown of false-positive predictions across the 4 thyroid-state groups within the negative class provides physiological context for the model’s classification behavior. Among the 1595 negative-class prediction outputs in the overall test cohort (single-reference configuration), the false-positive rate varied by underlying thyroid state: 38% (38/100) in subclinical hypothyroidism, 24.5% (148/603) in euthyroidism, 13.4% (92/687) in subclinical hyperthyroidism, and 14.1% (29/205) in overt hyperthyroidism. The rank ordering—subclinical hypothyroidism >euthyroidism>hyperthyroid states—is directionally consistent with the physiological premise that the model responds to reductions in circulating thyroid hormone, with elevated false-positive predictions among subclinical hypothyroid cases whose physiological trajectory partially overlaps with the overt hypothyroid target class and reduced false-positive predictions among hyperthyroid cases whose cardiovascular physiology is opposite in direction. However, we temper the interpretation of this pattern. From a classification standpoint, the same data also demonstrate substantial baseline misclassification among purely euthyroid observations (24.5%), and the subclinical excess over euthyroidism is a modest 13.5 percentage-point difference rather than the more dramatic contrast implied when hyperthyroid cases are pooled into the comparison baseline. This pattern reflects the inherent limitation of collapsing a continuous physiological spectrum into a binary positive or negative label at the free T4 threshold of 0.89 ng/dL, and it contributes directly to the reported PPV (21.1%). Explicit modeling of the subclinical transition zone as an intermediate class in larger, subclinical-enriched cohorts (as detailed in the *Future Work and Recommendations* section) remains an essential direction for reducing this class of misclassification and improving overall precision.

The PPV of 21.1% should be interpreted in the context of disease prevalence rather than as an isolated indicator of clinical utility. By the Bayes theorem, PPV is mathematically bounded above by the prior probability of disease in the evaluated population: with a hypothyroidism prevalence of 5.9% in the single-reference test cohort (100 positive out of 1695 prediction outputs), even a hypothetical classifier with 90% sensitivity and 90% specificity would yield a PPV of only approximately 36%. The observed PPV of 21.1% at a sensitivity of 82% and a specificity of 80.8% is therefore consistent with this prevalence-bounded behavior and reflects the expected operating characteristic of a well-calibrated triage tool applied to a low-prevalence target condition. For a secondary screening triage tool whose role is to prioritize confirmatory testing rather than to confirm diagnosis, the clinically more relevant operating characteristic is the NPV (98.6%), which directly quantifies the ability to safely defer unnecessary biochemical testing. In current practice, nonspecific symptoms such as fatigue often lead to frequent, unnecessary biochemical testing; by reliably ruling out hypothyroidism in a significant portion of these individuals [4,5], the system can reduce the overall volume of laboratory tests. We posit that the net health economic effect—the savings from avoiding unnecessary tests for the high-NPV group—outweighs the costs associated with confirmatory testing for false-positive cases, aligning with the demonstrated cost-effectiveness of digital thyroid management. Notably, recent studies have demonstrated that such AI-based monitoring tools can be cost-effective when used for thyroid dysfunction management in real-world clinical settings, supporting their utility not only in improving care quality but also in reducing overall health care expenditure [16,17].

### Limitations

Nevertheless, it is crucial to acknowledge several limitations of this study. First, the generalizability of our findings may be constrained by the demographic composition of our cohort, which comprised participants aged 20 to 60 years and lacked ethnic diversity. The hypothyroidism-related studies were restricted to this age range by design; the thyrotoxicosis-related studies permitted enrollment of adults aged >60 years, but no such participant was enrolled. We focused on this age range for initial validation, as it represents the demographic with the highest current adoption of wearable technology and digital health literacy, ensuring the data stability required to establish a reliable digital phenotype. Regarding ethnic diversity, extensive evidence suggests that the autonomic impact of thyroid dysfunction is physiologically preserved across different populations and even species [18-23]. This supports the premise that the physiological signals detected by our model are fundamentally conserved biological responses, suggesting that our system’s predictive performance may remain robust across different age groups and diverse human populations. Second, wearable-derived HR data are susceptible to external noise from psychological stress or lifestyle factors. We attempted to mitigate this by focusing on sleep HR, a period of relative autonomic stability, but some confounding physiological signals may remain. Third, this study used an iatrogenic model of acute hormone withdrawal to establish the initial digital HR phenotype. While this controlled approach was essential for precise identification of hormonal shifts, the physiological manifestations may differ from the more insidious onset of primary hypothyroidism. Consequently, further validation in naturally occurring cases will help fully define the system’s performance boundaries and ensure its optimal generalizability across the broader clinical spectrum of the disease. Furthermore, the feature selection procedure was conducted on the same dataset used for final evaluation, rather than being fully nested within each LOOCV fold. While participant-level separation was maintained throughout, a fully nested pipeline would represent a more rigorous approach and is recommended for future work. We acknowledge that this design introduces a nonzero risk of optimistic bias in the reported absolute performance metrics. Practically, the magnitude of this bias is expected to be modest because feature selection was driven by aggregate cross-validation AUC across the entire participant pool—so the exclusion of any individual participant has limited effect on the final selected feature set—and because the selected features represent established physiological descriptors with mechanistic links to autonomic function rather than dataset-specific artifacts. Nonetheless, the relative comparisons reported in the manuscript—including the relative performance gain with additional reference pairs, the more conservative subset analysis, and the complementary contribution of HR-derived features in the ablation analysis—are derived from configurations sharing this same design and are therefore expected to be more robust to uniform optimistic bias than the absolute metrics themselves. Unbiased estimation of absolute performance under strictly nested feature selection is identified as an essential methodological refinement for future prospective validation. Fourth, our data collection involved multiple wearable models and firmware versions, which may introduce measurement variances due to different proprietary algorithms. However, since our system relies on relative changes from an individualized baseline recorded by the same device, hardware-specific biases are largely mitigated. This approach reflects a “real-world” clinical monitoring environment where users may own various device generations. Within each study, all participants used a single designated device model throughout, and no device-switching occurred. However, we cannot rule out that firmware updates were applied during the data collection period, as Fitbit does not provide firmware version history through its API, and this was not systematically recorded in the original study protocols. This limitation is partially mitigated by the relative change-based feature design, which is inherently robust to device-specific systematic offsets. Fifth, the sex distribution of our cohort (3.17:1 female-to-male ratio) reflects, in a moderated form, the well-documented female predominance of hypothyroidism in population studies [4]. While this composition is clinically realistic for the intended deployment population, the small number of positive cases in the male subgroup (14 observations from 7 participants) precludes a statistically reliable evaluation of sex-specific sensitivity—the classification of even a single case shifts the estimate by approximately 7 percentage points. We note that established sex-related differences in resting HR distributions [20] are partially mitigated by the individualized baseline calibration of our system, which models relative physiological transitions rather than absolute HR values, conferring a degree of robustness against absolute sex-based HR differences. Nonetheless, a robust assessment of sex-based performance equity requires larger and more balanced cohorts with sufficient positive cases in both subgroups, which we identify as an important direction for future work. Sixth, the AI-assisted system uses a hard threshold–based routing mechanism (target mean sleep HR ≥62.5 bpm vs <62.5 bpm) to direct observations to 1 of 2 parallel submodels, which carries a theoretical risk of producing inconsistent predictions for participants whose mean sleep HR fluctuates around the boundary across repeated measurements. To empirically assess this risk, we analyzed the performance of observations falling within ±2 bpm of the boundary (60.5‐64.5 bpm; n=274, 16.2% of all observations), which achieved a sensitivity of 0.81 and specificity of 0.74—values comparable to the overall model performance (sensitivity 0.82 and specificity 0.81). This stability is attributable to the fact that observations near the boundary are present in both submodel training datasets, allowing each submodel to learn appropriate decision functions for the boundary region. Nonetheless, future implementations could explore soft routing mechanisms to further reduce this theoretical risk.

### Future Work and Recommendations

Future iterations should integrate multimodal biosignals—including skin temperature, electrodermal activity, and sleep architecture—to more effectively detect subtle physiological changes associated with thyroid dysfunction. Incorporating such multimodal data streams could yield a more comprehensive physiological profile and further improve the model’s sensitivity and specificity across the spectrum of thyroid dysfunction. Additionally, future multicenter external validation involving a broader age spectrum and diverse populations is essential to confirm the system’s robustness beyond a single-center setting and ensure its generalizability in real-world clinical practice. A natural methodological extension is to model the subclinical hypothyroidism transition zone as an explicit intermediate class rather than grouping it within the negative class; the limited number of subclinical observations in the present dataset (n=100) precludes statistically stable independent class modeling at this stage, motivating future cohorts enriched for subclinical hypothyroidism. Soft routing mechanisms—such as probability-weighted ensemble predictions based on distance from the submodel boundary threshold—could be explored to mitigate the theoretical risk of prediction instability at the hard-threshold decision boundary and to further refine model behavior in border-region observations. Real-world deployment will also require dedicated operational validation of alert protocols—including sequential decision rules that require consistent positive predictions across multiple consecutive sessions before triggering clinical follow-up—to empirically optimize the trade-off between false-positive burden and timely detection. Such protocol-level validation requires continuous daily prediction series paired with longitudinal ground-truth ascertainment across extended monitoring periods independent of discrete blood test dates, a deployment-evaluation framework methodologically distinct from the blood test–anchored prediction model evaluated in the present study.

### Conclusions

This study demonstrates that an AI system using wearable-derived sleep HR data can identify hypothyroid states, showing robust performance in both euthyroid-hypothyroid transitions and severe cases. These results suggest the feasibility of a noninvasive tool to prioritize individuals for confirmatory biochemical testing, particularly for those exhibiting significant physiological deviations. As validation was performed predominantly in female participants and in an iatrogenic-withdrawal cohort, prospective studies in sex-balanced populations with naturally occurring primary hypothyroidism will be required to establish real-world clinical performance.

## Supplementary material

10.2196/86030 Multimedia Appendix 1 State-wise test-cohort breakdown.

10.2196/86030 Multimedia Appendix 2 Reference-configuration counts for the euthyroid-hypothyroid subset (corresponding to Table 2) and the overall test cohort (corresponding to Table 3).

10.2196/86030 Multimedia Appendix 3 Input features of the AI-assisted hypothyroidism prediction system.

10.2196/86030 Multimedia Appendix 4 Benchmarking performance comparison of candidate classification algorithms under fair-comparison conditions.

## Abbreviations

AUC: area under the curve
AUPRC: area under the precision-recall curve
HR: heart rate
IRB: Institutional Review Board
LOOCV: leave-one-out cross-validation
NPV: negative predictive value
PPG: photoplethysmography
PPV: positive predictive value
PR: precision-recall
RAI: radioactive iodine
ROC: receiver operating characteristic
SHAP: Shapley Additive Explanations
SNUBH: Seoul National University Bundang Hospital
TFT: thyroid function test
TSH: thyroid-stimulating hormone
