# Supplementary material for: Harnessing AI for Hypothyroidism Monitoring Using Wearable-Derived Heart Rate Data: Retrospective Cohort Study
Source: JMIR AI. 2026 Oct 28;5:e86030. doi: 10.2196/86030 (PMC13716157; doi:10.2196/86030)
Supplement: Supplementary file 1 [file ai-v5-e86030-s001.docx]

**Multimedia Appendix 1. State-wise test-cohort breakdown.** The column "Test outputs" shows the number of prediction outputs contributed by each thyroid state under the single-reference configuration on the overall test cohort. Test data were restricted to observations with same-day sleep HR data on the target date and used only forward temporal orderings, reflecting the intended deployment scenario. The state assigned to each output is defined by the target-date thyroid function state. Because a single target date may pair with multiple prior reference dates, a target date can contribute more than one output; the 1,695 outputs arise from 753 unique target dates (1–14 reference pairings per target date; mean 2.25; 315 target dates had exactly one available reference). This multiplicity explains why the output counts of some states exceed the corresponding HR–TFT pair counts in Table 1. The total (1,695) matches the single-reference row of Table 3 (Overall Performance). The subset counts corresponding to Table 2 (Euthyroid–hypothyroid subset) are obtained by summing euthyroidism, subclinical hypothyroidism, and overt hypothyroidism rows (603 + 100 + 100 = 803, matching the single-reference row of Table 2); these 803 outputs arise from 352 unique target dates. A sensitivity analysis restricted to one output per unique target date is reported in the Results section of the main text. HR, heart rate; TFT, thyroid function test.

| Thyroid function state | Test outputs (n) |
| --- | --- |
| Overt hyperthyroidism | 205 |
| Subclinical hyperthyroidism | 687 |
| Euthyroidism | 603 |
| Subclinical hypothyroidism | 100 |
| Overt hypothyroidism | 100 |
| Total | 1,695 |
