# Supplementary material for: Harnessing AI for Hypothyroidism Monitoring Using Wearable-Derived Heart Rate Data: Retrospective Cohort Study
Source: JMIR AI. 2026 Oct 28;5:e86030. doi: 10.2196/86030 (PMC13716157; doi:10.2196/86030)
Supplement: Supplementary file 2 [file ai-v5-e86030-s002.docx]

**Multimedia Appendix 2. Reference-configuration counts for the euthyroid–hypothyroid subset (corresponding to Table 2) and the overall test cohort (corresponding to Table 3).** Under a k-reference configuration, the AI-assisted system uses k prior reference HR–TFT pairs from the same participant together with the target HR–TFT pair to generate a single prediction output for the target date; therefore, the number of reference–target pairs entering the model equals k × the number of outputs. As the number of required reference pairs increases, the number of participants meeting the eligibility criterion decreases (a k-reference output requires at least k+1 eligible HR–TFT pairs from the same participant), reducing the number of target predictions accordingly. The values in the "Prediction outputs" column correspond to the output-count values reported in Tables 2 and 3, and are the denominators of the performance metrics. HR, heart rate; TFT, thyroid function test.

| Test data cohort | Number of references | Reference–target pairs (n) | Prediction outputs (n) | Positive / Negative |
| --- | --- | --- | --- | --- |
| Euthyroid–hypothyroid subset (Table 2) | 1 | 803 | 803 | 100 / 703 |
|  | 2 | 408 | 204 | 25 / 179 |
|  | 3 | 315 | 105 | 11 / 94 |
| Overall cohort (Table 3) | 1 | 1,695 | 1,695 | 100 / 1,595 |
|  | 2 | 876 | 438 | 25 / 413 |
|  | 3 | 663 | 221 | 11 / 210 |
