# Supplementary material for: Harnessing AI for Hypothyroidism Monitoring Using Wearable-Derived Heart Rate Data: Retrospective Cohort Study
Source: JMIR AI. 2026 Oct 28;5:e86030. doi: 10.2196/86030 (PMC13716157; doi:10.2196/86030)
Supplement: Supplementary file 3 [file ai-v5-e86030-s003.docx]

| **Feature** | **Description** | **Mathematical Definition** |
| --- | --- | --- |
| **TFT (Laboratory) Features** | | |
| ref_fT4 | Reference serum free T4 level | Laboratory measurement (ng/dL) |
| **Statistical HR Features** | | |
| Arithmetic mean (target) | Mean of target sleep HR | mean = (1/n) · Σ xᵢ |
| Harmonic mean (target) | Harmonic mean of target sleep HR | n / Σ(1/xᵢ) |
| Harmonic mean (reference) | Harmonic mean of reference sleep HR | n / Σ(1/xᵢ) |
| Bottom 10% mean (target) | Mean of bottom 10th percentile of target sleep HR | mean({ xᵢ \| xᵢ ≤ Q₀.₁ }) |
| Skewness (target) | Skewness of target sleep HR distribution | Fisher-Pearson: (n/((n−1)(n−2))) · Σ((xᵢ−x̄)/s)³ |
| Binarized mean (target) | Proportion of target sleep HR values exceeding the mean | (1/n) · Σ 1(xᵢ > x̄) |
| Histogram mode (target) | Mode of target sleep HR histogram | Mode of 10-bin equal-width histogram |
| Kurtosis (reference) | Kurtosis of reference sleep HR distribution | Fisher excess kurtosis: [(n(n+1))/((n−1)(n−2)(n−3))] · Σ((xᵢ−x̄)/s)⁴ − [3(n−1)²/((n−2)(n−3))] |
| Variance heterogeneity (target) | Variance heterogeneity index of target sleep HR | Engle's ARCH LM statistic: n · R² |
| **Temporal HR Features** | | |
| Spikiness (target) | Spikiness index of target sleep HR | Var({ Var(R₋ᵢ) }), STL residual (period=20) |
| Seasonality strength (target) | Seasonality strength of target sleep HR | 1 − Var(Rₜ)/Var(Sₜ+Rₜ), STL (period=55) |
| Seasonal lag-1 ACF (target) | First seasonal autocorrelation of target sleep HR | ACF(x, lag=35), min. 72 observations |
| Seasonal lag-1 ACF (reference) | First seasonal autocorrelation of reference sleep HR | ACF(x, lag=35), min. 72 observations |
| Linearity (target) | Linearity index of target sleep HR time series | R² of linear regression fit: 1 − SSres/SStot |
| Level shift size (target) | Maximum level shift magnitude of target sleep HR | maxₖ \|mean(Wₖ₊₁) − mean(Wₖ)\|, window=45 min |
| SD of 1st differences (target) | Standard deviation of first differences of target sleep HR | std({ xᵢ₊₁ − xᵢ \| i = 1,...,n−1 }) |
| SD of 1st differences (reference) | Standard deviation of first differences of reference sleep HR | std({ xᵢ₊₁ − xᵢ \| i = 1,...,n−1 }) |
| Spectral entropy (target) | Normalized spectral entropy of target sleep HR | −∫ f̂(λ) · log f̂(λ) dλ |
| Trend strength (target) | Trend strength of target sleep HR | 1 − Var(Rₜ)/Var(Tₜ+Rₜ), STL (period=30) |
| KPSS unit root statistic (target) | KPSS test statistic of target sleep HR | (1/(n²·σ̂²)) · Σ Sₜ², regression='ct', nlags=1 |
| **Change and Rate Features (Derived from Above)** | | |
| *change_[feature]* | Absolute change between reference and target time points | tgt_[feature] − ref_[feature] |
| *change_[feature]_rate* | Relative change between reference and target time points | (tgt_[feature] − ref_[feature]) / \|ref_[feature]\| |

**Multimedia Appendix 3. Input features of the AI-assisted hypothyroidism prediction system.** Features are extracted from 1-minute resolution Fitbit sleep HR data recorded over 15 consecutive nights. All HR features are computed independently at the reference (ref) and target (tgt) time points, where ref denotes the reference blood test date and tgt denotes the target (prediction) date. Change features (change_[feature]) represent the absolute difference between time points; rate features (change_[feature]_rate) represent the relative change normalized by the reference value. The first table defines the candidate feature families from which model inputs were drawn. The second table enumerates the 26 features constituting the final curated input set retained after feature selection, indicating for each feature its derivation and the submodel(s) in which it is used (P submodel: target mean sleep HR ≥ 62.5 bpm, 19 features; M submodel: target mean sleep HR < 62.5 bpm, 14 features; 7 features are shared by both submodels). Feature names follow the labels used in Figure 3 of the main text. HR: heart rate; TFT: thyroid function test; STL: Seasonal-Trend decomposition using LOESS; ACF: autocorrelation function; KPSS: Kwiatkowski-Phillips-Schmidt-Shin; SD: standard deviation.

| **Feature (as labeled in Figure 3)** | **Derivation** |
| --- | --- |
| **Features used in both submodels (n = 7)** | |
| ref_fT4 | Laboratory feature (reference serum free T4) |
| Variance heterogeneity (target) | Base feature, target date |
| Harmonic mean change | change_[Harmonic mean] |
| Seasonality strength rate change | change_[Seasonality strength]_rate |
| Arithmetic mean rate change | change_[Arithmetic mean]_rate |
| Linearity change | change_[Linearity] |
| Kurtosis rate change | change_[Kurtosis]_rate |
| **Features used in the P submodel only (n = 12)** | |
| Spikiness (target) | Base feature, target date |
| Seasonal lag-1 ACF (target) | Base feature, target date |
| Seasonality strength (target) | Base feature, target date |
| Seasonal lag-1 ACF (reference) | Base feature, reference date |
| Kurtosis (reference) | Base feature, reference date |
| Harmonic mean rate change | change_[Harmonic mean]_rate |
| SD of 1st differences rate change | change_[SD of 1st differences]_rate |
| Linearity rate change | change_[Linearity]_rate |
| Trend strength rate change | change_[Trend strength]_rate |
| Level shift size rate change | change_[Level shift size]_rate |
| KPSS unit root change | change_[KPSS unit root statistic] |
| Arithmetic mean change | change_[Arithmetic mean] |
| **Features used in the M submodel only (n = 7)** | |
| Skewness (target) | Base feature, target date |
| Binarized mean (target) | Base feature, target date |
| Bottom 10% mean rate change | change_[Bottom 10% mean]_rate |
| SD of 1st differences change | change_[SD of 1st differences] |
| Variance heterogeneity change | change_[Variance heterogeneity] |
| Histogram mode rate change | change_[Histogram mode]_rate |
| Spectral entropy change | change_[Spectral entropy] |
