# Supplementary material for: Harnessing AI for Hypothyroidism Monitoring Using Wearable-Derived Heart Rate Data: Retrospective Cohort Study
Source: JMIR AI. 2026 Oct 28;5:e86030. doi: 10.2196/86030 (PMC13716157; doi:10.2196/86030)
Supplement: Supplementary file 4 [file ai-v5-e86030-s004.docx]

**Multimedia Appendix 4. Benchmarking performance comparison of candidate classification algorithms under fair-comparison conditions.** Benchmarking was conducted under participant-level group 10-fold cross-validation using the full pool of 107 candidate features (without prior feature selection), with balanced class weighting applied to all algorithms. Each algorithm was configured with its standard hyperparameters and was permitted to leverage its own internal feature weighting and regularization mechanisms (L1/L2 regularization for logistic regression; node-splitting criteria for random forest; gradient-based boosting for LightGBM). This benchmarking framework was designed for relative algorithm comparison under fair conditions and is therefore not directly comparable to the LOOCV-based primary analysis with the curated 26-feature set (Tables 2 and 3), which uses a different cross-validation protocol and feature space dimensionality. AUROC, area under the receiver operating characteristic curve; AUPRC, area under the precision–recall curve.

| Model | AUROC | AUPRC |
| --- | --- | --- |
| LightGBM | 0.695 | 0.118 |
| Random Forest | 0.687 | 0.112 |
| Logistic Regression | 0.664 | 0.139 |
